# A cohort study of immune and hematopoietic functionality changes in severe aplastic anemia patients treated with immunosuppressive therapy

**DOI:** 10.1097/MD.0000000000014149

**Published:** 2019-01-18

**Authors:** Jing Guan, Yingying Sun, Rong Fu, Huaquan Wang, Erbao Ruan, Xiaoming Wang, Wen Qu, Guojin Wang, Hong Liu, Yuhong Wu, Jia Song, Limin Xing, Lijuan Li, Hui Liu, Chunyan Liu, Zonghong Shao

**Affiliations:** Department of Hematology, Tianjin Medical University General Hospital, Tianjin, China.

**Keywords:** anemia, aplastic, hematopoietic functionality, immune functionality, T-cell

## Abstract

To investigate if variations in immune and hematopoietic parameters correlated with immunosuppressive therapy (IST) in severe aplastic anemia (SAA) patients.

A total of 115 SAA patients who received IST were included. Their immune and hematopoietic functionality changes had been evaluated at 0, 0.5, 1, 2, and 3-year(s) IST.

For SAA patients with complete remission (CR), the CD4^+^/CD8^+^T cell ratio continued to increase after a year of IST. The T helper (Th)1/Th2 ratio continued to decrease after 6 months of IST, as did the activated CD8^+^ T cell percentage. The myeloid dendritic cell (mDC)/plasmacytoid dendritic cell (pDC) ratio after 3 years of IST was significantly lower compared to that of untreated patients. The mDC/pDC and Th1/Th2 ratios exhibited positive correlation. The activated CD8^+^ T cell percentage and the number of peripheral blood neutrophils showed inverse correlation. For SAA patients with partial remission (PR), the CD4^+^T cell percentage increased at 1-year post-IST, but the later changes were not statistically significant. The other immune indexes of patients in partial remission group and nonremission (NR) group showed no obvious recovery. For all SAA patients, the percentage of T regulatory cells in CD4^+^ lymphocyte was higher in post-IST group compared to the pretreatment group.

For SAA patients responded well to IST, increase in peripheral neutrophils and improvement in bone marrow myeloid cells were first observed followed reduction in the activated CD8^+^ T cell percentage, Th1/Th2 ratio, CD4^+^/CD8^+^T ratio, along with mDC/pDC ratio, all of which negatively correlated with the hematopoietic parameters. This demonstrates that IST prompts improvements of hematopoietic functionalities of the SAA patients by regulating their immune functionalities.

## 1 Introduction

T-lymphocyte cell functional abnormalities are strongly associated with the occurrence, development, and prognosis of aplastic anemia.^[1]^ Many studies have shown that the mDC counts are abnormally high in aplastic anemia (AA) patients, which leads to polarization of Th cells that skews the ratio of Th1 to Th2 heavily toward the former. Excessive Th1 cell secretion of Type I lymphocyte causes an increase in the number of activated CD8^+^ effector T-cells, spurs apoptosis of bone marrow hematopoietic cells, and triggers hematopoietic dysfunction and pancytopenia.^[2]^

An effective targeted treatment strategy for AA is yet to be established due to a lack of understanding of its initiation and pathogenesis. Hence, understanding the changes in immune functionality of the antigen activated SAA patients will be beneficial in designing a long-term effective treatment. We attempted to prolong patient survival period before the antigen is naturally depleted, thus achieving a hematological remission progress, avoiding recurrence and cloning development, with the hope of finding an eventual cure for AA.

In this study, we selected 115 SAA patients, all of whom had received IST. Several immune and hematopoietic parameters were monitored before the start of treatment as well as at different times during the course of the treatment. The goal of this study was to explore the remission patterns of the immune and hematopoietic functionalities of the CR patients, as well as the correlations among these parameters. These results can be considered as the theoretical basis for establishing scientific and effective AA treatment plans and early prognosis of SAA.

## 2 Materials and methods

### 2.1 Cases

A total of 115 SAA patients, who had received IST, were selected in this study. They were treated in the Hematology Department of Tianjin Medical University General Hospital from October 2004 to July 2014. SAA diagnostic procedures were compliant with the 2009 British Council for Standardization in hematology aplastic anemia treatment guidelines.^[3]^ Treatment efficacy was determined based on the Camitta standard published in 1979.^[4]^ CR was achieved in 31 patients, of which 20 were male, while 39 cases showed PR and 45 patients did not respond to IST. The median age of the CR patients was 25 years, with the age range being 6 to 62. The follow-up duration of the 31 CR patients ranged from 6 to 60 months, with the median was at 12 months. In detail, one case had a 6-month follow-up period, 12 cases had a 12-month follow-up period, 10 cases had a 24-month follow-up period, while the follow-up periods of the remaining eight patients were beyond 36 months (see Table 1). The study was approved by the Ethics Committee of the Tianjin Medical University. Informed written consent was obtained from all patients or their guardians in accordance with the Declaration of Helsinki.

**Table 1:**
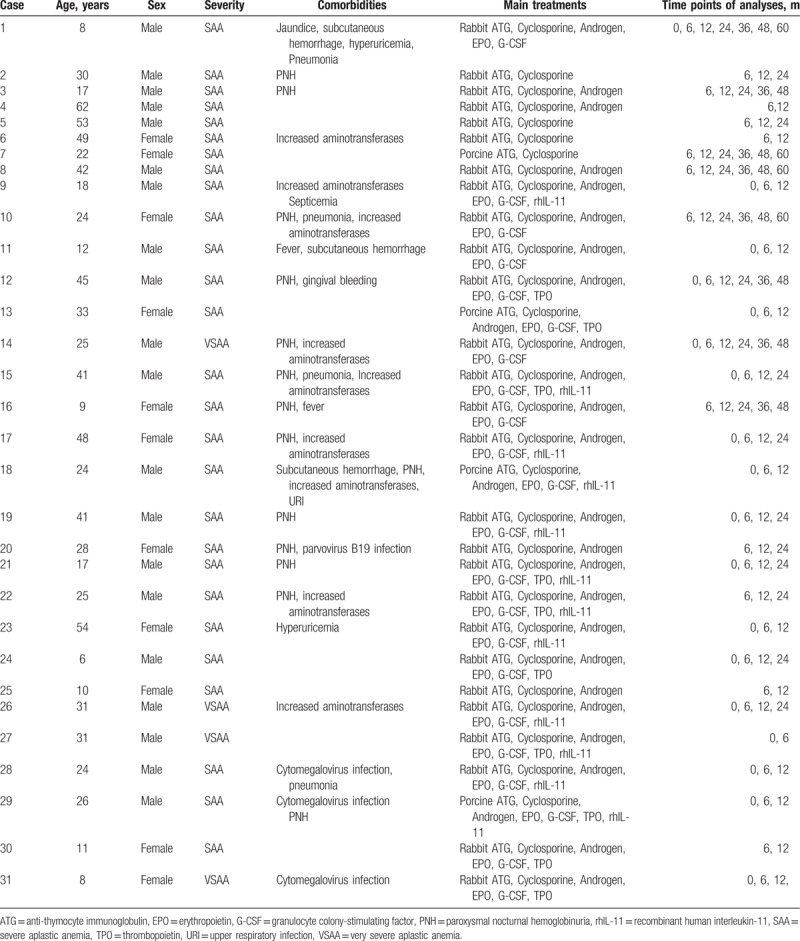
Characteristics of SAA patients achieved complete remission after IST.

### 2.2 Treatment plan

After diagnosis, all patients had received IST including anti thymocyte immunoglobulin (ATG)/or antihuman lymphocyte globulin (ALG), and cyclosporine. In the CR group, the rabbit ATG was used in 27 cases, with a dose of 3.75 mg/kg body weight for a total of 5 days. The porcine ATG was used in four cases, with a dose of 20 to 30 mg/kg body weight for a total of 5 days. The dose for cyclosporine is 3 to 5 mg/kg body weight. All patients were also treated with hematopoietic growth factors (HGFs). Medication was stopped for six patients who maintained CR after the treatment.

### 2.3 Immune and hematopoietic parameters

Immune parameters monitored included the DC subset, Th cell subset, T regulatory cell subset, T-cell subset and activated CD8^+^ T-cell percentage; while the hematopoietic parameters included the peripheral blood neutrophils and reticulocyte counts, bone marrow granulocytes to erythroid ratio as well as bone marrow megakaryocyte count. These parameters were measured at 0, 0.5, 1, 2, 3-year(s) IST (The peripheral blood platelet count was excluded since it was greatly affected by the blood transfusion).

### 2.4 Flow cytometric analysis

Peripheral blood T-cell subset, bone marrow DC, Th cell, T regulatory cell subset and activated CD8^+^ T-cells were measured by flow cytometry at the time of diagnosis, using the following directly labeled antibodies:

- T-cell subset: anti-CD3-PerCP, anti-CD4-FITC, anti-CD8-PE.
- DC subset: anti-CD123-PE, anti-CD11c-PE. mDC is CD11c^+^; pDC is CD123^+^.
- Th cell subset: anti-IFN-γ-PE, anti-IL-4-PE, anti-CD4-FITC. Th1 cell is CD4^+^ IFN-γ^+^; Th2 cell is CD4^+^ IL-4^+^.
- Activated CD8^+^ T-cell: anti-CD8-PE, anti-HLA-DR-PerCP.
- T regulatory cell subset: anti-CD4-FITC, anti-CD25-PE, anti-CD127-PerCP.

All the monoclonal antibodies were products of BD Pharmingen.

### 2.5 Statistical analysis

Patient data were analyzed using the SPSS 17 statistical analysis software. Results were expressed as mean ± standard deviations. The independent sample mean comparison had been done using the *t*-test (for data with normal distribution) and nonparametric test (for data without normal distribution). Spearman's test was used for determining linear correlation of the data. A value of *P < *.05 was considered statistically significant.

## 3 Results

### 3.1 Change in peripheral blood T subset

As shown in Table 2, the percentage of CD3^+^CD4^+^ cells started to increase after a year of IST (*P < *.05) in the CR group compared to that before treatment. The percentage of CD3^+^CD8^+^ cells began to gradually decrease after 2 years of IST (*P < *.05). The ratio of CD3^+^CD4^+^/CD3^+^CD8^+^ cells showed moderate increase after 1 year of IST (*P < *.05) and were significantly higher at 2 and 3 years of IST. The ratio approached to 1 after 3 years IST (normal value is 1.66 ± 0.33).

**Table 2:**
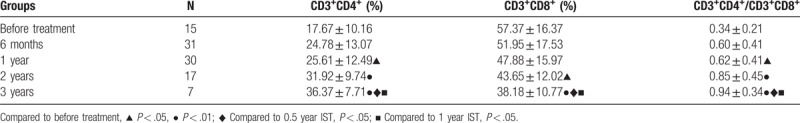
Changes of peripheral blood CD3^+^CD4^+^, CD3^+^CD8^+^ percentage and ratio in complete remission group.

In SAA patients who showed partial remission (PR), the CD4^+^ T-cell percentage increased after a year post-IST (29.29 ± 9.80 vs 18.13 ± 9.92, *P = *.04), but later changes were not statistically significant. The other immune indexes of patients in PR group and NR group showed no obvious recovery.

### 3.2 Changes in the bone marrow Th cell percentage and ratio

As shown in Table 3, in CR group, there is no statistical difference in the bone marrow Th1 and Th2 percentage before and after the IST. The ratio of Th1/Th2 cells gradually decreased at 6 months of IST. There was no statistical difference among Th cell data measured at different time points in the PR and NR groups.

**Table 3:**
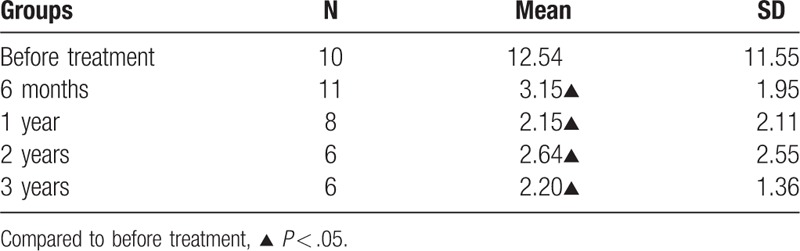
Change of Th1 and Th2 ratio in complete remission group.

### 3.3 Changes in the bone marrow mDC and pDC percentage and ratio

The CR patients were divided into five groups according to the measurements taken at 0, 0.5, 1, 2, 3 years of IST. There was no statistical difference in the bone marrow mDC and pDC percentage among groups. In the 3-year group, the ratio of bone marrow mDC and pDC (n = 12 with median = 0.08 and quartile = 1.06) was significantly lower than that before the commencement of treatment (n = 13 with median = 2.67 and quartile = 3.44) (*P < *.05). In the PR and NR groups, there was no significant change in mDC percentage, pDC percentage, and mDC/pDC ratio before and after IST.

### 3.4 Change in the bone marrow activated CD8^+^T-cell percentage

The activation state of the cytotoxic T-cells was expressed as the percentage of CD8^+^HLA-DR^+^ in CD8^+^ cells (CD8^+^HLA-DR^+^/CD8^+^). As manifested in Table 4, the percentage of CD8^+^HLA-DR^+^ started to gradually decrease at 6 months IST in CR group (*P < *.05).

**Table 4:**
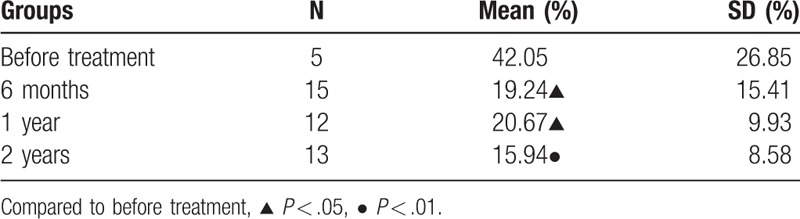
Change of bone marrow activated CD8^+^T cell percentage in complete remission group.

### 3.5 Change in T regulatory cell percentage

For all SAA patients, the percentage of T regulatory cells in CD4^+^ lymphocyte was higher in the post-IST group (4.23 ± 1.36, n = 26) than that in pretreatment group (3.07 ± 0.75, n = 21) (*P < *.05). The data of the above two groups were both lower than that of the normal control group (4.99 ± 1.34, n = 31), but only the pretreatment group and the normal control group showed statistical differences (*P < *.05).

### 3.6 Changes in hematopoietic parameters

#### 3.6.1 Change in hemogram

For all CR patients, measured at 0.5,1,2,3 year(s), the counts of peripheral blood neutrophil and reticulocyte significantly increased compared to those before treatment (*P < *0.01), as shown in Table 5.

**Table 5:**
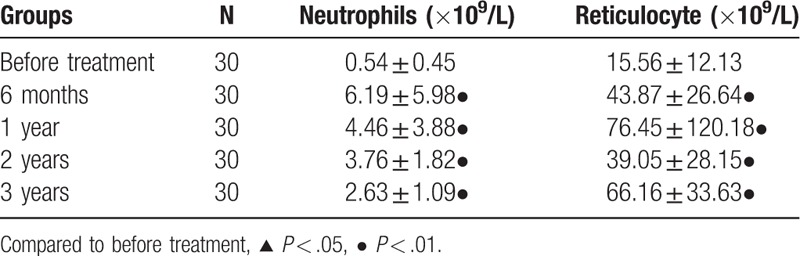
Changes of peripheral blood neutrophils and reticulocyte counts in complete remission group.

#### 3.6.2 Change in bone marrow image

The ratio of bone marrow granulocytes and erythrocytes as well as the quantity of megakaryocyte in CR group were analyzed in the study participants. After the treatment, bone marrow granulocytes percentage, erythrocytes percentage and megakaryocyte count gradually increased in all patients in comparison to those before treatment (*P < *.05), as shown in Table 6.

**Table 6:**
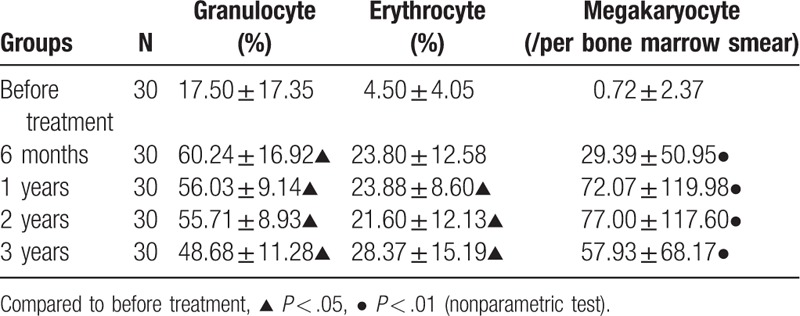
Changes of bone marrow granulocyte percentage, erythrocyte percentage, and megakaryocyte count in complete remission group.

### 3.7 Correlations of immune and hematopoietic parameters

#### 3.7.1 Correlation of immune parameters

**Figure 1:**
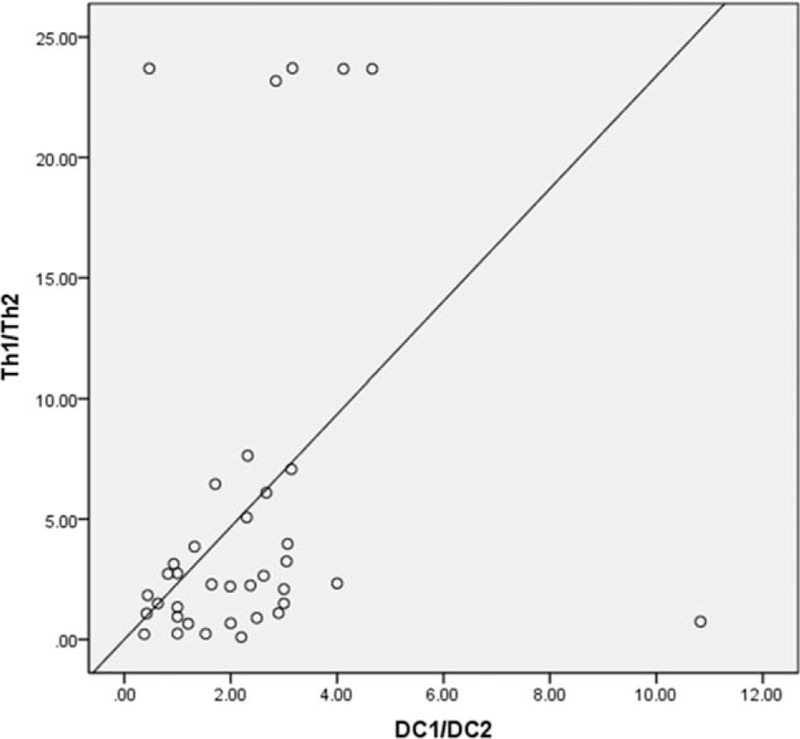
Positive correlation between mDC/pDC ratio and activated CD8^+^ T-cell percentage.

As shown in Figure 1, the mDC/pDC ratio correlated with that of Th1/Th2 (*r* = 0.36, *P < *.05) for CR patients. The mDC/pDC ratio and activated CD8^+^ T-cell percentage also exhibited a positive correlation (*r* = 0.32, *P < *.05). The ratio of CD4^+^/CD8^+^ and percentage of activated CD8^+^ T-cells were inversely correlated (*r* = 0.32, *P < *.05).

#### 3.7.2 Correlation of immune and hematopoietic parameters

**Figure 2:**
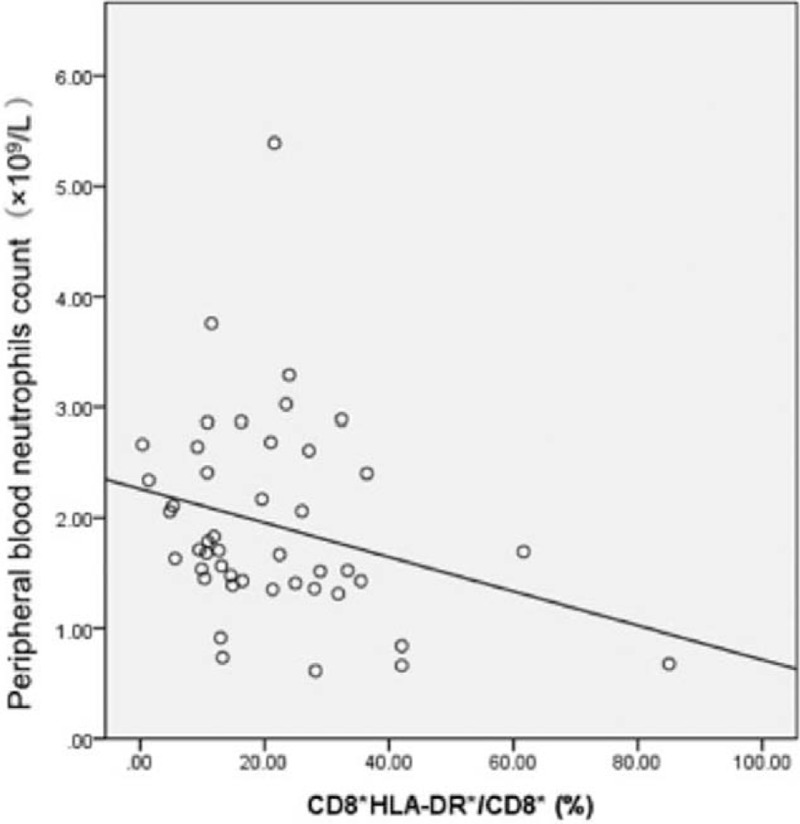
Inverse correlation between activated CD8^+^ T-cell percentage and peripheral blood neutrophil count.

As shown in Figure 2, we observed an inverse correlation between the percentage of activated CD8^+^ T-cells and peripheral blood neutrophil count (*r* = −0.32, *P < *.05) in the CR group.

## 4 Discussion

Aplastic anemia (AA) is an autoimmune disease that is mediated by an increased number of dysfunctional T lymphocytes. Previous studies have reported that some antigenic substances trigger the activation of mDCs,^[5]^ resulting in a dramatic increase of the mDC/pDC ratio,^[6]^ increased secretion of T-bet, Th0 polarization to Th1 causing the ratio of Th1/Th2 to be skewed in favor of Th1.^[7]^ These Th1 cells secrete a copious amount of interferon (INF)-γamong other cytokines, which is a negative regulator hematopoietic progenitor colonies, mediates cell immunity, activates cytotoxic T-cells (CD8^+^ T-cells), and causes a reduction in the CD4^+^/CD8^+^ ratio^[8]^ by increasing the percentage of activated CD8^+^ T-cells.^[9]^ The CD8^+^ T-cells are the key effector cells in AA pathogenesis that enable the apoptosis of bone marrow hematopoietic cells mediated by perforin, Granzyme B and Fas/FasL,^[10]^ resulting in bone marrow failure. The aforementioned immune functionality changes can be described as an “immune activation waterfall” that results in AA pathogenesis.

IST has been effective in 60% to 70% of AA cases and has become the treatment of choice for SAA patients.^[11]^ Approximately, 60% of the SAA patients showed improvement between 3 and 6 months after commencing IST. At this point, the patient condition was monitored predominantly by the hematopoietic parameters.^[12]^ In order to infer a common pattern of changes in the immune functionality of SAA patients before and after commencement of IST, a deeper understanding of the pathogenesis was required, which in turn helped us gain insights on the occurrence and development of the disease. In order to ensure the homogeneity of the follow-ups, the studied cases were selected based on the effectiveness of the treatment. Multiple immune parameters reflecting T-cell functionality and quantity were measured and analyzed to reveal the immune functionality dynamics with respect to time.

Our study results showed a dramatic improvement in all hematopoietic parameters of the CR patients at 6 months post-IST. However, changes in the immune parameters were gradual and in a chronological order which suggests a possible pattern in the development of the disorder. Activated CD8^+^ T-cell percentage first dipped at 6 months post-IST, suggesting the activation of the cytotoxic T-cells was inhibited. Concomitantly, the ratio of Th1/Th2 decreased dramatically, indicating a possibility of reduction of Th1-mediated cytotoxicity. A year after IST, along with the decrease of activated CD8^+^T-cell percentage and Th1/Th2 ratio, we also observed a significantly increase in CD4^+^ while CD8^+^ was markedly reduced. At 2 years after IST, the improvement of the above-mentioned three parameters was all statistically significant suggesting that the number of cytotoxic T-cells decreased after IST, following the inhibition of cytotoxic T-cell activation. At 3 years after commencement of IST, the above-mentioned immune parameters continued to improve and the mDC/pDC ratio decreased significantly compared to that before treatment (*P < *.05). Among all the immune parameters analyzed, this is chronologically the last immune cell subset to show improvement. The dendritic cells (DC) are key antigen presenting cells that are upstream of the AA “immune activation waterfalls.” T lymphocytes are stimulated by antigen signals from DC. The binding of the MHCs and costimulatory molecules expressed by DCs with the molecules on the surface of T-cells initiates secretion of multiple cytokines that regulate T-cell response and immune tolerance. The correlation between the ratios of mDC/pDC and Th1/Th2 further manifested the direct relationship of DC and Th subset, an important link in the T “waterfall activation” during the pathogenesis of AA. The positive correlation between the mDC/pDC ratio and activated CD8^+^ T-cell percentage indicated that the changes in quantity and functionality of DC subset were somehow related to the activation of cytotoxic T-cells. The inverse correlation between CD4^+^/CD8^+^ ratio and activated CD8^+^ T-cell percentage suggested that T subset inversion and increase of CD4^+^/CD8^+^ ratio and the activation of cytotoxic T-cells were correlated. The CD4^+^/CD8^+^ ratio increased with the inhibition of cytotoxic T-cell activation. The inverse correlation between the percentage of activated CD8^+^ T-cells and the number of peripheral blood neutrophils indicated that the activation of the cytotoxic T-cells could damage the hematopoietic functionality of the bone marrow. Eventually, the count of mature cells in the peripheral blood decreased. Since IST alleviated the damage caused to the blood cells, the neutrophil count increased and cytotoxic T cell activation decreased. Based on the above correlation analysis, the improvement in percentage and ratios of various immune cell subsets were associated for SAA patients who were treated with IST. These eventually led to a return to normal blood routine, that is, hematologic CR of SAA. In PR and NR groups, the hematopoietic parameters did not increase sufficiently possibly due to a lack of sufficient improvement in the immune parameters.

The effect of IST on the immune parameters proved a critical factor in our study for complete recovery and long-term recovery in aplastic anemia. These observations emphasize on steps to monitor on immune parameters alongside the hematopoietic improvements to effectively treat patients.

Our study had several limitations. Firstly, the sample size was relatively small, particularly in the CR group. Several patients were not available in latter follow-up times. Our future efforts will be focused on incorporating more AA patients to achieve a larger sample size and thus improved statistics.

## 5 Conclusion

In summary, we show that the improvement of immune parameters in AA patients by immunosuppressive therapy could lead to complete recovery and the improvement of the immune parameters was critical for long-term recovery from aplastic anemia.

## Author contributions

**Conceptualization:** Jing Guan, Yingying Sun, Zonghong Shao.

**Data curation:** Jing Guan, Yingying Sun, Huaquan Wang, Zonghong Shao.

**Formal analysis:** Jing Guan, Yingying Sun, Huaquan Wang, Zonghong Shao.

**Funding acquisition:** Jing Guan, Yingying Sun, Zonghong Shao.

**Investigation:** Jing Guan, Yingying Sun, Rong Fu, Huaquan Wang, Erbao Ruan, Xiaoming Wang, Wen Qu, Guojin Wang, Hong Liu, Yuhong Wu, Jia Song, Limin Xing.

**Methodology:** Jing Guan, Yingying Sun.

**Project administration:** Jing Guan, Yingying Sun.

**Resources:** Jing Guan, Yingying Sun, Lijuan Li, Hui Liu, Chunyan Liu.

**Software:** Jing Guan, Yingying Sun, Lijuan Li, Hui Liu, Chunyan Liu.

**Supervision:** Jing Guan, Yingying Sun.

**Validation:** Jing Guan, Yingying Sun.

**Visualization:** Jing Guan, Yingying Sun.

**Writing – original draft:** Jing Guan, Yingying Sun, Zonghong Shao.

**Writing – review & editing:** Jing Guan, Yingying Sun, Zonghong Shao.

Yingying Sun orcid: 0000-0002-0788-7076.
